# Sentiment Analysis of Social Media on Childhood Vaccination: Development of an Ontology

**DOI:** 10.2196/13456

**Published:** 2019-06-07

**Authors:** Jeongah On, Hyeoun-Ae Park, Tae-Min Song

**Affiliations:** 1 College of Nursing, Seoul National University Seoul Republic of Korea; 2 Research Institute of Nursing Science, Seoul National University Seoul Republic of Korea; 3 Department of Health Management, Sahmyook University Seoul Republic of Korea

**Keywords:** social media, vaccination, health information interoperability, semantics

## Abstract

**Background:**

Although vaccination rates are above the threshold for herd immunity in South Korea, a growing number of parents have expressed concerns about the safety of vaccines. It is important to understand these concerns so that we can maintain high vaccination rates.

**Objective:**

The aim of this study was to develop a childhood vaccination ontology to serve as a framework for collecting and analyzing social data on childhood vaccination and to use this ontology for identifying concerns about and sentiments toward childhood vaccination from social data.

**Methods:**

The domain and scope of the ontology were determined by developing competency questions. We checked if existing ontologies and conceptual frameworks related to vaccination can be reused for the childhood vaccination ontology. Terms were collected from clinical practice guidelines, research papers, and posts on social media platforms. Class concepts were extracted from these terms. A class hierarchy was developed using a top-down approach. The ontology was evaluated in terms of description logics, face and content validity, and coverage. In total, 40,359 Korean posts on childhood vaccination were collected from 27 social media channels between January and December 2015. Vaccination issues were identified and classified using the second-level class concepts of the ontology. The sentiments were classified in 3 ways: positive, negative or neutral. Posts were analyzed using frequency, trend, logistic regression, and association rules.

**Results:**

Our childhood vaccination ontology comprised 9 superclasses with 137 subclasses and 431 synonyms for class, attribute, and value concepts. *Parent’s health belief* appeared in 53.21% (15,709/29,521) of posts and positive sentiments appeared in 64.08% (17,454/27,236) of posts. Trends in sentiments toward vaccination were affected by news about vaccinations. Posts with *parents’ health belief*, *vaccination availability*, and *vaccination policy* were associated with positive sentiments, whereas posts with *experience of vaccine adverse events* were associated with negative sentiments.

**Conclusions:**

The childhood vaccination ontology developed in this study was useful for collecting and analyzing social data on childhood vaccination. We expect that practitioners and researchers in the field of childhood vaccination could use our ontology to identify concerns about and sentiments toward childhood vaccination from social data.

## Introduction

Vaccination is an effective means of inducing active immunity against infection by administering a vaccine made by killing or weakening the pathogenicity of microorganisms. In addition to preventing individuals from becoming ill, this creates herd immunity, thus preventing transmission of infection through social groups [1]. Childhood vaccination, starting at infancy when the immune system is weak, is very important for immunogenesis and disease prevention. Therefore, the government of each country sets out schedules for childhood vaccinations and recommends vaccination at the appropriate time. The Korean Centers for Disease Control and Prevention (KCDC) recommends that parents vaccinate their children aged younger than 12 years according to a standard vaccination schedule and supports vaccination with policies, such as the provision of financial support [2].

According to a national childhood vaccination coverage survey conducted by KCDC in 2016, the vaccination rate of children aged 3 years was 96.5% [3], which is higher than the herd immunity threshold. However, there are concerns raised by the public that vaccination might not be effective in preventing infectious disease, especially when there were intermittent outbreaks of vaccine-preventable diseases, such as viral hepatitis A and varicella in South Korea. The public questions about the safety of vaccines because of bogus rumors, such as the Measles, Mumps, and Rubella (MMR) vaccines causing autism and thimerosal, a mercury-based preservative contained in some vaccines, causing brain damage [4].

The number of posts on social media claiming that vaccination is not necessary or even harmful is increasing. These negative sentiments toward vaccination may affect people’s intention to vaccinate and thus lead to a reduction in vaccination rates [5-9]. Therefore, to maintain vaccination rates above the herd immunity threshold, it is necessary to monitor public concerns about and sentiments toward vaccination and identify factors affecting them.

Several studies have assessed public knowledge and perceptions of vaccination, intent to vaccinate, and factors affecting vaccination intent or behavior [5-8,10-12]. Most of these studies were conducted using mail or telephone surveys or personal interviews. However, there are disadvantages of these survey studies, such as long research time, small sample size, representativeness of sample, low response rates, and interviewer bias.

Social networking services (SNSs) are emerging as a medium that can be used to identify public concerns and sentiments in various fields [13]. Social data are used to identify concerns about vaccination, such as their safety and side effects [14-16], as well as sentiments toward vaccines in general [14] or toward specific vaccines, such as the MMR vaccine [17], influenza A vaccination [18], and Human Papilloma Virus (HPV) vaccine [19]. In addition, social data are used to identify correlations between sentiments toward vaccines and epidemics of vaccine-preventable diseases [17,18] and vaccine information flow [18]. In South Korea, no research has yet examined concerns about and sentiments toward vaccines and vaccination using social data.

The internet usage rate of Koreans is as high as 90.3% and 68.2% of them use social media [20]. Specifically, the usage rate of those aged 20 to 30 years who can be parents of young children, is as high as 89.5% [20]. In fact, there are about 45,000 parenting communities in the online cafes of the 2 most popular Web portals in South Korea, Daum and Naver. Thus, data on childhood vaccination can be obtained, and vaccination issues and sentiments can be identified. Vaccination issues in this study were defined as opinions, perceptions, concerns, and worries about vaccines and vaccination posted on social media. Sentiments in this study were defined as feeling and emotion on childhood vaccination posted on social media.

Social media posts are unstructured data composed mostly of text. To use such unstructured data for analysis, a hierarchical classification of terms, relationships of terms, such as synonyms and hyponyms, and clustering based on the frequency of terms are used [21,22]. However, these methods are not sufficient for understanding the semantics of terms [21,22]. An ontology defining the meanings and inherent attributes of concepts, capturing relationships between them, and containing terms covering thesaurus, is required for social data analysis to solve this issue [21,23-25]. An ontology can help researchers understand the semantics of and the relationships between concepts when contextual knowledge is lacking. Terms included in the ontology help researchers collect the social data that appear in the form of various synonyms. Unfortunately, no ontology is yet available that can be used to identify vaccination issues and sentiments toward childhood vaccination.

Therefore, we developed an ontology for use as a framework for collecting and analyzing social data on childhood vaccination. We used this ontology to identify vaccination issues and sentiments toward childhood vaccination, trends in these sentiments, and relationships between vaccination issues and sentiments in social data posted in Korean.

## Methods

### Development of a Childhood Vaccination Ontology

We developed a childhood vaccination ontology by following the Ontology Development 101 [26]. Ontology development consists of the following 5 steps, and it was an iterative process.

#### Step 1: Determining the Domain and Scope of the Ontology

To determine the domain and scope of the ontology, we first created competency questions [27], which is a list of questions that the childhood vaccination ontology should be able to answer, for example: *What are the childhood vaccination items?* The competency questions were also used for ontology evaluation in the final step.

#### Step 2: Consideration of Reuse of Existing Ontologies

We searched for existing ontologies and conceptual frameworks related to vaccination in BioPortal [28], a repository of biomedical ontologies and research papers. We identified the Vaccine Ontology [29], which contains classifications of vaccines and vaccine components, vaccine quality and phenotypes, and host immune responses to vaccines. We also identified 5 models of vaccination decisions and hesitancy [30-34]. They are as follows: (1) a conceptual model of the role of parental attitudes and beliefs in decision making about child and adolescent vaccination [30], (2) a conceptual framework for HPV vaccine acceptance and adherence, focusing on sociocultural factors impacting vaccine adherence behavior [31], (3) a framework for determinants of H1N1 influenza vaccine uptake utilizing the social ecological model [32], (4) a model for assessing determinants of vaccine hesitancy in different settings [33], and (5) a conceptual model of determinants of individual decision making about vaccination [34]. We reviewed whether the Vaccine Ontology and the models of vaccination decisions and hesitancy could be used to develop a childhood vaccination ontology.

#### Step 3: Collecting Terms and Extracting Concepts

We collected terms within the domain and scope of the ontology by reviewing 3 vaccination practice guidelines developed by the KCDC [35], the United States Centers for Disease Control and Prevention [36], and the Public Health England [37], and 101 research papers found by searching for the keywords *vaccination* and *immunization*. In addition, we searched SNSs to identify new terms that were not collected from practice guidelines or research papers. We extracted class concepts from the terms.

#### Step 4: Developing the Ontology and Terminology

The class hierarchy was developed using a top-down approach. The superclasses of the ontology and their relationships were constructed by integrating 2 models of vaccination decisions and hesitancy [31,32]. Subclasses were defined by grouping of the vaccination-related concepts from the 5 models of vaccination decisions and hesitancy [30-34]. The extracted concepts were arranged by mapping or adding to the class hierarchy. We represented class concepts as entity-attribute-value (EAV) models. We also developed a terminology, including synonyms for class, attribute, and attribute value concepts. The ontology was developed using the Protégé 5.0.0 ontology editor.

#### Step 5: Evaluating the Ontology

We evaluated the ontology with description logic (DL) verification, face and content validity, and coverage evaluation. DL of ontology was verified by whether the ontology provided appropriate answers to the competency questions developed earlier. We converted competency questions into Protégé DL queries (DL-query) using the class concepts and relationships between classes and tested whether it generated the correct answer upon entering a DL-query. For example, the competency question, *What are the adverse events experienced after vaccination?* was converted to the DL-query, *hasType some experience of vaccine adverse event*. After entering this query into Protégé, we tested whether the answers to the query, such as *edema* and *hypersensitivity*, were adverse events associated with the vaccine.

Face validity of the ontology was evaluated by 3 health informatics experts in biomedical ontology. They were asked to assess superficially and subjectively assess whether the ontology is valid for identifying childhood vaccination issues. Content validity of ontology was evaluated by 3 domain experts with Master’s degree and more than 4 years of experience in pediatric nursing. They were asked to rate the ontology classes on a 4-point scale (1=very invalid, 2=invalid, 3=valid, and 4=very valid) as to whether it is valid for identifying childhood vaccination issues. The content validity index (CVI) of the ontology was calculated by taking the average of the class-level CVIs, which were computed by dividing the number of experts with 3 to 4 points by the total number of experts for each class.

Coverage of the ontology was examined by comparing terms extracted from social data with the concepts and synonyms of the ontology. We revised the ontology by adding new terms.

### Analyzing Social Data on Childhood Vaccination

We collected posts on childhood vaccination posted in the Korean language from 27 social media channels between January 1 and December 31, 2015 (see Multimedia Appendix 1). The channels included 1 SNS (Twitter, a popular microblogging site), 2 online cafes (Daum Café and NAVER Café, 2 popular online community services), 4 internet blogs (eg, the NAVER blog), and 20 message boards (eg, NATE Pann). We used *vaccination* as a search keyword and *vaccine injection*, *child vaccination*, *child vaccine injection*, *infant vaccination*, *infant vaccine injection*, *toddler vaccination*, and *toddler vaccine injection* as synonyms. Posts on livestock or plant vaccination were excluded. Data were collected by Smart Insight [38], a big-data marketing platform. The social data do not have any identifiable personal information, such as user profiles. This study was approved by the Institutional Review Board of the Korea Institute for Health and Social Affairs.

We preprocessed the collected social data by treating the analysis unit as a single post. After extracting the terms from each post, we identified the terms related to vaccination as vaccination issues and the emotional words as sentiments. The terms identified as vaccination issues were classified into the second-level classes of the ontology. The emotional words identified as sentiments were classified into positive and negative emotional words using a *universal emotion keyword list* developed by Smart Insight. We counted the numbers of positive and negative emotional words in each post. When the number of positive emotional words was more than the number of negative emotional words, the post was classified as a positive sentiment. When the number of negative emotional words was more than the number of positive emotional words, the post was classified as a negative sentiment. When the number of positive emotional words was equal to the number of negative emotional words, the post was classified as a neutral sentiment.

We analyzed the frequencies of vaccination issues and sentiments. We compared monthly sentiment trends with selected vaccination-related news. Vaccination-related news was selected from news articles on vaccines, vaccination, and infectious diseases, searched from Naver, which is one of the largest news portals in South Korea. We performed sentiment analysis by conducting logistic regression analysis and association analysis. We used logistic regression analysis to investigate how vaccination issues affect sentiments. Significant vaccination issues from univariate analyses (*P*<.05) were used as independent variables and sentiments as the dependent variable, with positive and negative sentiments converted to *1* and *0*, respectively. We used association analysis applying a priori principle algorithm to investigate which sets of vaccination issues were associated with sentiments. The interestingness of the association rules are expressed as support, confidence, and lift. Support means proportion of posts with set of vaccination issues and sentiments in the entire posts. Confidence means proportion of posts with sentiments among posts with set of vaccination issues. Lift means ratio of probability of sentiments when set of vaccination issues is appeared to probability of sentiments when set of vaccination issues is not appeared. We restricted the consequences of rules to positive and negative sentiments. A frequent item set was defined as rules satisfying minimal support (0.05) and confidence (0.5) constraints. We used the R software package (version 3.3.1) for both logistic regression and association analyses.

## Results

### Development of a Childhood Vaccination Ontology

We determined the domain and scope of the ontology from personal perceptions, behavior, and experiences of vaccination as well as social, environmental, institutional, and political factors related to vaccination based on 21 competency questions (see Textbox 1) created. We restricted vaccinations to those recommended by the KCDC for children aged 0 to 12 years.

A total of 5 models of vaccination decisions and hesitancy [30-34] were used in the childhood vaccination ontology development. We modified the domain and scope of the ontology by renaming personal perceptions as parent factor, and adding child factor, family and friend factor, organizational factor, and community factor related to vaccination.

We collected 883 terms covering the domain and scope of ontology and extracted 133 unique class concepts from these terms. We defined hierarchical and attribute relationships between classes based on 5 models of vaccination decisions and hesitancy [30-34]. Various factors affect vaccine uptake, decision making, and hesitancy [30,32-34]. Vaccination experience is also an important factor affecting completion of the multistep vaccination schedule [31]. We viewed vaccination as a process progressing from *before vaccination* and *vaccination* onto *after vaccination*. Based on the Social Ecological Framework for H1N1 Influenza Vaccine Uptake in the United States [32], we placed *child*, *parent*, *family and friend*, *organization and institution*, *society and community*, and *policy* levels of factors affecting vaccination before vaccination. We also placed *vaccination intention* before vaccination. *Vaccination behavior* was placed at vaccination and *vaccination experience* was placed after vaccination. Various levels of factors affect vaccination intention, behavior, and experience. Vaccination intention affects vaccination behavior, and vaccination behavior affects vaccination experience. Vaccination experience, in turn, affects vaccination intention for the next round of vaccinations.

**A list of competency questions.**

What are the childhood vaccination items?

Where is the child vaccinated?

How many vaccinations does it take?

Which body sites are vaccinated?

When should vaccination be appropriate?

What is the cost of vaccination?

Do parents know about vaccination?

Where do parents get vaccination information?

What are the vaccination policies?

What influences vaccination decisions?

What do parents think about the child’s side when deciding on vaccination?

Which parents vaccinate their child?

What are the parents’ health belief that influence vaccination decisions?

What previous vaccination experience influenced vaccination decisions?

What are the family and friend side factors that influence vaccination decisions?

What agencies are involved in vaccination or vaccine?

What are the epidemics affecting vaccination decisions?

What are the media reports that influence vaccination decisions?

What are the adverse events experienced after vaccination?

What is the experience of medical staff involved in vaccination?

What are the sentiments about vaccination?

**Figure 1:**
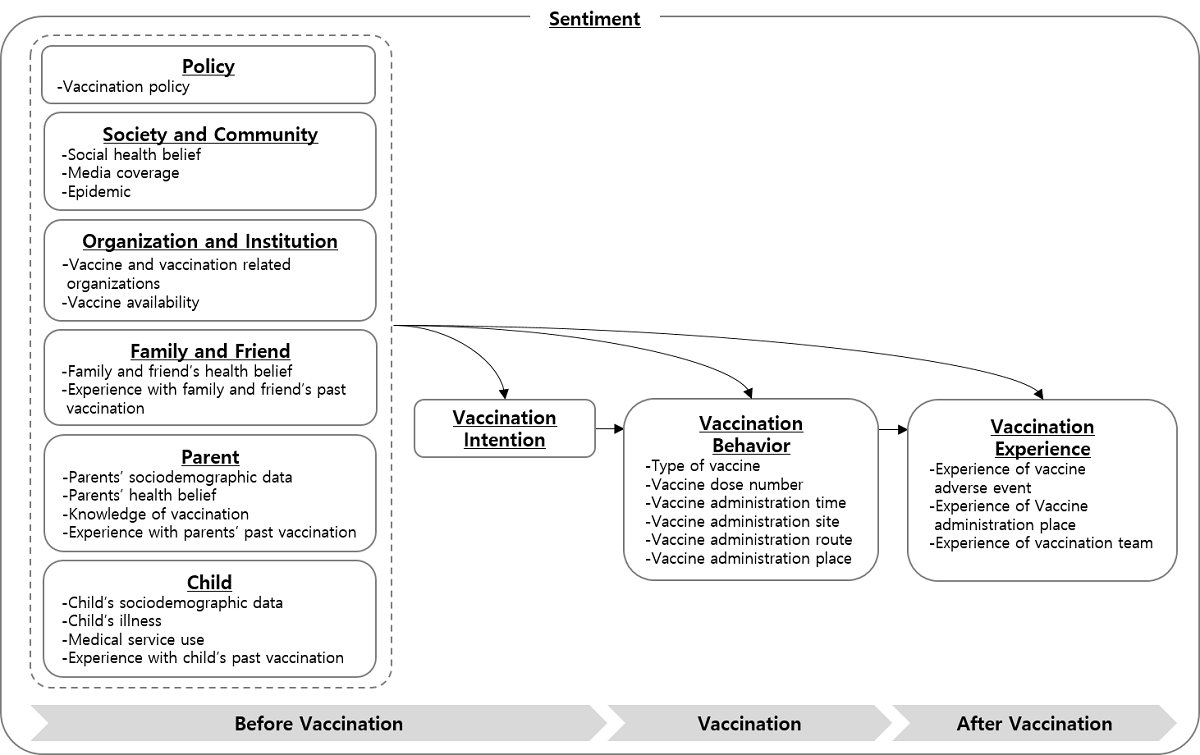
Childhood vaccination ontology up to the second-level class.

Figure 1 shows the childhood vaccination ontology (up to the second level) that we developed. The ontology consists of 9 superclasses: *child, parent, family and friend, organization and institution, society and community, policy, vaccination intention, vaccination behavior,* and *vaccination experience*. *The child* class included child-related factors for vaccination, such as age, illness, and experience with past vaccination. *The parent* class included parent-related factors for vaccination, such as health belief and knowledge about vaccination. *The family and friend* class included family- and friend-related factors for vaccination, such as family and friend’s health belief. *The organization and institution* class included vaccine- and vaccination-related organizations and vaccine availability. *The society and community* class included media coverage and epidemics. *The policy* class included vaccination policy. *The vaccination intention* class included decisions on whether to vaccinate in future. *The vaccination behavior* class included the types and vaccine dose number of the vaccination administered. *The vaccination experience* class included experience after vaccination, such as adverse events. The depths of the superclasses varied from 1 level for *vaccination intention* to 4 levels for *parent*.

We developed EAV models of the 103 lowest level class concepts. For example, *experience of vaccine adverse event* had attributes of *hasType* and *has* *S* *everity*. Of these, *hasType* had values, such as *pain* and *edema*, and *has* *S* *everity* had values, such as *mild*, *moderate*, and *severe*. We also developed a terminology composed of 126 synonyms for 133 classes, 1 synonym for 12 attributes, and 343 synonyms for 268 values.

With the DL verification, it was found that the ontology correctly answered all 21 competency questions. With the face validity, it was found that the ontology was superficially and subjectively valid for identifying childhood vaccination issues. With the content validity, it was found that the CVI of the ontology was 1.0 and all classes of the ontology were valid for identifying childhood vaccination issues. With coverage evaluation, 138 of the 148 (93.2%) concepts were covered by the ontology and 163 out of 575 (28.3%) synonyms were covered by the ontology. We revised the ontology by adding 10 value concepts, 288 synonyms for classes, and 124 synonyms for values. Finally, the childhood vaccination ontology was composed of 133 classes, 414 synonyms for 133 classes, 1 synonym for 12 attributes, and 467 synonyms for 278 values.

### Analyzing Social Data on Childhood Vaccination

#### Frequency Analysis of Vaccination Issues and Sentiments Toward Vaccination

We collected 40,359 posts on childhood vaccination. Vaccination issues were grouped into 17 second-level classes of ontology. Table 1 shows frequencies of each vaccination issue in the posts. *Parents’ health belief* appeared in 15,709 out of 29,521 posts (53.21%) and *experience of vaccine administration place* appeared in 14,964 out of 29,521 posts (50.69%). The lowest frequency vaccination issue was *vaccination intention*, which appeared in 198 out of 29,521 posts (0.67%). Sentiments toward vaccination appeared in 27,236 out of 40,359 posts (67.48%). Positive sentiments appeared in 17,454 out of 27,236 posts (64.08%), negative sentiments appeared in 7121 out of 27,236 posts (26.15%), and neutral sentiments appeared in 2661 out of 27,236 posts (9.77%).

**Table 1:** Frequency of vaccination issues (total posts, N=29,521).

Vaccination issues	n (%)
Parents’ health belief	15,709 (53.2)
Experience of vaccine administration place	14,964 (50.7)
Epidemic	13,386 (45.3)
Experience of adverse event	10,125 (34.3)
Medical service use	9002 (30.5)
Child’s illness	8512 (28.8)
Type of vaccine	8191 (27.7)
Vaccination availability	8011 (27.1)
Knowledge of vaccination	6774 (22.9)
Experience of vaccination team	5310 (18.0)
Media coverage	5050 (17.1)
Vaccine dose number	5037 (17.1)
Vaccine- and vaccination-related organizations	4886 (16.6)
Vaccination policy	4235 (14.3)
Vaccine administration site	2077 (7.0)
Vaccine administration time	753 (2.6)
Vaccination intention	198 (0.7)

#### Trend Analysis of Sentiments Toward Vaccination

**Figure 2:**
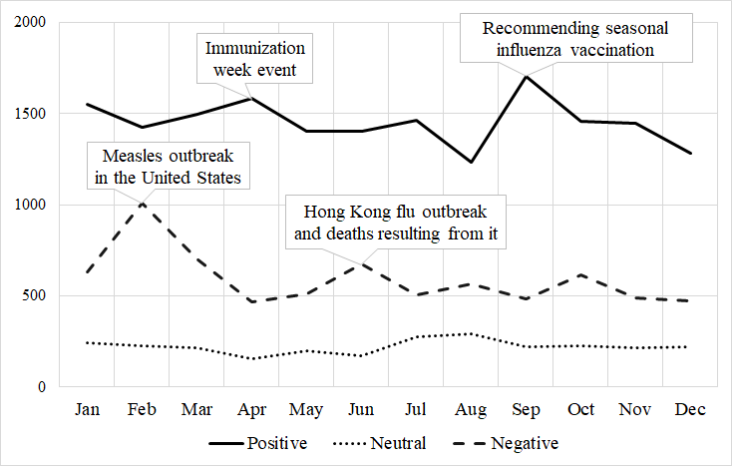
Monthly sentiment trends and vaccination related news.

Monthly sentiment trends with vaccination-related news are shown in Figure 2. Positive sentiments increased in April and September, and negative sentiments increased in February and June. Neutral sentiments showed little variation. In February, when negative sentiments increased, the news about the spread of measles in the United States had been reported. In April, when positive sentiments increased, the news about vaccination weekly events had been reported. In June, when negative sentiments increased, the news about the spread of Hong Kong flu and associated deaths had been reported. In September, when positive sentiments increased, the news about the KCDC recommendations on seasonal influenza vaccinations had been reported.

#### Sentiment Analysis

The results of multivariate analysis of vaccination issues on sentiments over time are shown in Table 2. Before vaccination, *vaccination policy*, *parents’ health belief*, and *vaccination availability* affected positive sentiments; *child’s illness* and *knowledge of vaccination* affected negative sentiments. The time of vaccination, *vaccine administration time*, *vaccine dose number*, and *vaccine administration site*, affected positive sentiments. After vaccination, *experience of vaccine adverse event* affected negative sentiments and *experience of vaccine administration place* affected positive sentiments. The results of a multivariate analysis of vaccination issues on sentiments are shown in Table 3. *Vaccine administration time*, *vaccination policy*, *parents’ health belief*, *vaccination availability*, *vaccine dose number*, and *vaccine administration site* affected positive sentiments. *Experience of vaccine adverse event*, *child’s illness*, and *knowledge of vaccination* affected negative sentiments.

**Table 2:** Multivariate analyses of vaccination issues on sentiment over time.

Time and vaccination issues		Estimate	SE	z value	*P* value	Odds ratio
**Before vaccination**						
	Vaccination policy	0.80	0.05	15.22	<.001	2.23
	Parents’ health belief	0.65	0.03	19.63	<.001	1.91
	Child’s illness	-0.37	0.03	-10.74	<.001	0.69
	Vaccination availability	0.29	0.04	8.02	<.001	1.34
	Knowledge of vaccination	-0.25	0.04	-6.89	<.001	0.78
	Vaccination intention	0.19	0.21	0.92	.36	1.21
	Vaccine- and vaccination-related organizations	-0.03	0.04	-0.61	.54	0.98
**Vaccination**						
	Vaccine administration time	0.87	0.13	6.88	<.001	2.38
	Vaccine dose number	0.38	0.04	9.23	<.001	1.46
	Vaccine administration site	0.17	0.06	2.98	<.001	1.19
**After vaccination**						
	Experience of vaccine adverse event	-0.16	0.03	-5.21	<.001	0.85
	Experience of vaccine administration place	0.14	0.03	4.65	<.001	1.15

**Table 3:** Multivariate analysis of vaccination issues on sentiment.

Vaccination issues	Estimate	SE	z value	*P* value	Odds ratio
Vaccinated administration time	0.72	0.13	5.62	<.001	2.06
Vaccination policy	0.72	0.05	13.41	<.001	2.04
Parents’ health belief	0.70	0.03	20.31	<.001	2.01
Experience of vaccine adverse event	-0.33	0.04	-8.53	<.001	0.72
Vaccination availability	0.30	0.04	7.89	<.001	1.35
Vaccine dose number	0.29	0.04	6.84	<.001	1.34
Child’s illness	-0.27	0.04	-6.81	<.001	0.76
Vaccine administration site	0.24	0.06	3.93	<.001	1.27
Knowledge of vaccination	-0.23	0.04	-6.25	<.001	0.79
Experience of vaccine administration place	-0.04	0.03	-1.33	.18	0.96
Vaccination intention	0.03	0.21	0.16	.88	1.03
Vaccine- and vaccination-related organizations	0.00	0.04	-0.02	.99	1.00

**Table 4:** Association rules with top 5 lift and bottom 5 lift.

Rules	Support	Confidence	Lift
Health belief^a^, availability^b^, policy^c^ ⇒ Positive sentiment	0.06	0.92	1.29
Availability, policy, place^d^ ⇒ Positive sentiment	0.05	0.90	1.27
Availability, policy ⇒ Positive sentiment	0.07	0.89	1.25
Availability, place, organizations^e^ ⇒ Positive sentiment	0.06	0.89	1.25
Health belief, policy ⇒ Positive sentiment	0.09	0.89	1.25
Health belief, knowledge^f^, place, AE^g^ ⇒ Positive sentiment	0.05	0.57	0.80
Knowledge, place, AE ⇒ Positive sentiment	0.06	0.57	0.81
Health belief, illness^h^, AE ⇒ Positive sentiment	0.06	0.59	0.83
Health belief, knowledge, AE ⇒ Positive sentiment	0.07	0.59	0.83
Knowledge, AE ⇒ Positive sentiment	0.07	0.60	0.84

^a^Parents’ health belief.

^b^Vaccination availability.

^c^Vaccination policy.

^d^Experience of vaccine administration place.

^e^Vaccine- and vaccination-related organizations.

^f^Knowledge of vaccination.

^g^AE: experience of vaccine adverse event.

^h^Child’s illness.

The results of analysis of sets of vaccination issues associated with sentiments are shown in Table 4.

Of the 80 rules, Table 4 shows the 5 rules with the highest lift and the 5 rules with the lowest lift. For example, the sets of *parents’ health belief*, *vaccination availability*, and *vaccination policy* were associated with positive sentiments with support of 0.06, confidence of 0.92, and lift of 1.29. A support level of 0.06 indicates that 6% of posts have *parents’ health belief*, *vaccination availability*, and *vaccination policy* along with positive sentiments. A confidence level of 0.92 indicates that 92% of posts with *parents’ health belief*, *vaccination availability*, and *vaccination policy* have positive sentiments. A lift level of 1.29 indicates that the ratio of appearance of positive sentiments in posts with *parents’ health belief*, *vaccination availability*, and *vaccination policy* to the appearance of positive sentiments in total posts is 1.29. The set of *parents’ health belief*, *knowledge of vaccination*, *experience of vaccine administration place*, and *experience of vaccine adverse event* was associated with positive sentiments with a lift of 0.80. This indicates that the ratio of appearance of positive sentiments in posts with *parents’ health belief*, *knowledge of vaccination*, *experience of vaccine administration place*, and *experience of vaccine adverse event* to the appearance of positive sentiments in total posts is 0.80. In other words, the ratio of appearance of negative sentiments in posts with these vaccination issues to the appearance of negative sentiments in total posts is 1.25.

## Discussion

### Principal Findings

We developed a childhood vaccination ontology as a framework for systematically collecting and analyzing social data on childhood vaccination and used this ontology to identify public concerns about and sentiments toward childhood vaccination from social data.

The childhood vaccination ontology developed in this study had the following characteristics. First, this ontology was the first ontology describing childhood vaccination, including factors affecting vaccination, as well as vaccination intention, behavior, and experience. Although there was a preexisting Vaccine Ontology [29] available, it was not suitable for identifying public concerns about and sentiments toward childhood vaccination from social data. Second, this ontology included various factors affecting vaccination, such as *child, parent, family and friend, organization and institution, society and community,* and *policy*, by modifying the Social Ecological Framework [32]. This ontology was not limited to factors affecting vaccination but also included intention before vaccination, vaccination behavior, and experience after vaccination. Third, this ontology included Is-A and attribute relationships among concepts. Classes were modeled as EAVs and thus included attributes of each class and values of the attributes. Finally, this ontology included terminology with synonyms for classes, attributes, and values of attributes so that we can collect and analyze the social media posts on childhood vaccination.

According to frequency analysis, the most common vaccination issue was *parent’s health belief* (53.2%). This was also one of the important topics in other surveys on vaccination decision making and hesitancy [5,7,8,12,30,31,39]. Positive sentiments appeared 2.5 times more than negative sentiments. Positive sentiments toward vaccination were also identified more often than negative sentiments in other studies based on social data [14,18,40]. Members of the public who consider vaccination to be a useful measure to prevent infectious diseases might post narratives with positive sentiments.

According to a trend analysis of sentiments, public sentiments toward vaccination fluctuated with news about vaccination. Positive sentiments increased when news encouraging vaccination, such as vaccination campaigns, was announced, whereas negative sentiments increased when news about epidemic outbreaks, such as the measles outbreak in the United States, was announced. Other studies have also identified increases in vaccine-related posts, including positive or negative sentiments, to news stories about vaccination [41,42].

According to logistic regression analysis, the public who are aware of aspects of vaccination policy, such as promotion of free vaccinations and the increasing number of medical institutions offering free vaccinations, and public with health belief that vaccination is preventing infectious disease and such diseases are serious if not prevented, posted positive sentiments whereas the public who experienced unwanted adverse events after vaccination posted negative sentiments. Therefore, it is important to inform the public of vaccination policy, the benefits of vaccination, and how to deal with adverse events to lower negative sentiments, which can affect vaccination intention or behavior.

According to association analysis of vaccination issues with sentiment, the public who posted vaccination policy and vaccination availability in terms of cost and distance to appropriate medical institutions posted more positive sentiments whereas the public who posted adverse event experienced after vaccination and knowledge of vaccination posted more negative sentiments. This was also found in logistic regression analysis. *Parents’ health belief* and *experience of vaccine administration place* were associated with both positive and negative sentiments. These were posted in more than half of analyzed posts in which both positive and negative sentiments appeared.

### Limitations

We classified the sentiments of the posts by comparing the numbers of words expressing positive and negative emotions. We did not reflect weight and degree of emotion, such as severe and mild, and did not distinguish double negatives or tense of words when classifying sentiments. There are other studies used machine learning algorithms [17,42] to classify the emotions in social data or a sentiment score [19] that expresses sentiments as a numerical value. We suggest developing and using new emotion classification algorithm reflecting weight and degree of emotion or sentiment score as further research.

We were not able to identify yearly trends in sentiments and vaccination issues because of the short duration of data collection period. We suggest studying yearly sentiments trends using data collected from more than 1 year in future research.

We were not able to study vaccination intention or behavior because of a lack of posts discussing these topics. Vaccination intention and behavior can be studied by combining various data sources, such as survey data and existing vaccination statistics from the immunization registry in the future.

We used only those vaccination issues classified in terms of the second-level class concepts of the ontology developed in this study. We suggest applying lower-level class concepts and their relationships, for example, different types of vaccination and types of adverse events, in future research.

Use of social data for research is justified because of public accessibility of social media data. However, there exist ethical concerns around privacy and the protection of sensitive information, because it involves collecting data involving human subjects [43]. The public might stop posting their concerns or opinions on vaccination-related issues on social media if they realize that their posts are being analyzed by data scientists. To solve these issues, we anonymized social data by removing identifying information and did not use any social media quotes that might identify a social media user.

### Conclusions

In this study, we developed a childhood vaccination ontology comprising 9 superclasses and 124 subclasses with 4 levels of depth and a terminology containing 882 synonyms for class, attribute, and value concepts. We used this ontology as a framework to identify the public concerns about and sentiments toward childhood vaccination from social data. This is the first study to analyze public concerns about and sentiments toward childhood vaccination using social media posts by developing an ontology.

*Parent’s health belief*, *vaccination availability*, and *vaccination policy* were the 3 most significant factors associated with positive sentiment. Health belief may be influenced by antivaccine arguments such as the view that natural immunity is better than vaccine-acquired immunity or baseless rumors claiming that vaccines cause autism. Thus, it is important to monitor antivaccine arguments and rumors posted on the social media that might increase negative sentiment toward vaccination. *Vaccination availability* including cost and the travel distance to vaccine administration place is related to *vaccination policy,* such as the increasing number of free vaccines and number of health care institutions offering free vaccinations. Thus, it is important to publicize policies on free vaccinations to improve positive sentiments toward vaccination. As negative sentiments toward vaccination affect people’s intention to vaccinate and thus lead to a reduction in vaccination rates [5-9], it is important to introduce ways to improve positive sentiments toward vaccination.

We expect that practitioners and researchers in the field of childhood vaccination may use this ontology to identify public concerns about and sentiments toward childhood vaccination from social data.

## Appendix

Multimedia Appendix 1 Social media channels: a list of social media channels used to collect social data.

## Abbreviations

CVI: content validity index
DL: description logic
EAV: entity-attribute-value
HPV: Human Papilloma Virus
KCDC: Korean Centers for Disease Control and Prevention
MMR: Measles, Mumps, and Rubella
NRF: National Research Foundation of Korea
SNS: Social Networking Service
